# Effect of a Baduanjin intervention on the risk of falls in the elderly individuals with mild cognitive impairment: a study protocol for a randomized controlled trial

**DOI:** 10.1186/s12906-023-04050-4

**Published:** 2023-07-13

**Authors:** Ziyi Wu, Yuxing Kuang, Yiwen Wan, Jiao Shi, Shuqian Li, Rui Xia, Mingyue Wan, Shangjie Chen

**Affiliations:** 1 The Second School of Clinical Medicine, Southern Medical University, Guangzhou, 510515 China; 2 Department of Rehabilitation,The People’s Hospital of Baoan Shenzhen, The Second School of Clinical Medicine, Southern Medical University, Shenzhen, 518101 China; 3 Shunde Maternal and Children’s Hospital, Guangdong Medical University, Foshan, 528300 China

**Keywords:** Baduanjin, Falls, MCI, Randomized controlled trial, RCT protocol

## Abstract

**Background:**

Falls are a global public problem and may be an important cause of death in older adults. However, older adults with mild cognitive impairment(MCI) are more likely to fall and suffer more damage than older adults with normal cognitive function, which shows the importance of preventing falls. More and more evidence shows that Baduanjin can improve the balance function of the elderly and reduce the risk of falls in the elderly with MCI, but the mechanism is still unclear. The main purpose of this study is to verify the intervention effect of Baduanjin training on the risk of falls in elderly people with MCI and to elucidate the underlying mechanism of Baduanjin training in reducing the risk of falls in MCI patients.

**Methods:**

In this prospective study, outcome assessor-blind, three-arm randomized controlled trial, a total of 72 eligible participants will be randomly allocated (1:1:1) into the 12-week Baduanjin exercise intervention (60 min per session, three sessions per week), the 12-week brisk walking group(60 min per session, three sessions per week) or the 12-week health education group. Primary outcome is the Fall-Risk Self-Assessment Questionnaire(FRQ), and secondary outcomes are fall efficacy index, gait assessment, balance function, lower limb muscle strength, cognitive function, activities of daily living(ADL) and MRI scans. In addition to the MRI scans, which will be measured before and after the intervention,other primary and secondary outcomes will be assessed at baseline, 6 weeks, and 12 weeks (at the end of the intervention) and after an additional 12-week follow-up period. The mixed linear model will be conducted to observe the intervention effects.

**Discussion:**

This trial will investigate the effect of Baduanjin exercise on the prevention of falls in elderly individuals with MCI, explore the imaging mechanism of Baduanjin exercise to reduce the risk of falls in elderly individuals with MCI from the perspective of vestibular neural network, and provide strong evidence for Baduanjin exercise to reduce the risk of falls in elderly individuals with MCI, as well as provide new ideas and approaches for the central mechanism of Traditional Chinese Medicine(TRC) rehabilitation methods to intervene in falls in elderly.

**Trial registration:**

Chictr.org.cn, ID: ChiCTR2200057520. Registered on 14 March 2022, https://www.chictr.org.cn/showproj.html?proj=146592.

## Introduction

### Background

According to data from the World Health Organization, falls account for over 38 million disability-adjusted life years lost each year and are the second greatest cause of unintentional injury deaths after traffic accidents [1]. Approximately 15% of people over 65 years old had fallen at least once in the past 12 months [2]. Falls in elderly individuals are a leading cause of death [3] and can result in serious injuries such as fractures, joint dislocations, sprains, strains, and concussions [4]. Older persons who have MCI are at a higher risk of falling [5, 6]. Compared to older people with normal cognitive function, MCI increases the incidence of falls by more than two times [7, 8], and hip fractures after a fall are three times more common [9]. This shows that older people with MCI are at higher risk of falls, are facing a more hazardous situation, and require focused research and attention.

The mechanisms by which falls occur are complex, with the vestibular nerve pathway playing a key role. External receptors transmit signals via the vestibular nerve to the vestibular nucleus and cerebellum in the corresponding brainstem, where they are integrated and processed with other sensory information (e.g. visual information, other proprioceptive information). Multiple neural pathways and central loops transmit information to higher levels of the brain, where it is encoded and processed for decision-making, and ultimately the motor output system sends nerve impulses to control the skeletal muscle system to maintain balance and postural correction [10]. The vestibular circuit is plastic and enhancing sensory input and neuromuscular response activity can improve vestibular function, thereby reducing the risk of falls.

According to a new study published in JAMA, exercise interventions are the most effective way to lower the risk of falls in older persons [11]. A Cochrane systematic review involving 23,407 participants from 108 randomized controlled trials has provided strong evidence that exercise is helpful in reducing falls in community-dwelling older adults [12]. Due to the decline in physical strength and cognitive function in elderly individuals with MCI, it is not suitable to perform high-intensity, complex, or high-agility movements. Baduanjin is an exceptional representative from of traditional Chinese sports therapy, with the characteristics of harmony of body and mind and mutual support of body and spirit. Baduanjin movements are simple and suitable for elderly individuals with MCI to practice, and Baduanjin is practiced by a diverse group of individuals. Studies suggest that Baduanjin can significantly improve memory, executive function, and processing speed in older people [13, 14]; it can also improve balance, leg strength, and mobility and reduce the risk of falls [15–17].However, the mechanism by which Baduanjin reduces the risk of falls in MCI patients remains unclear.

The mechanism by which Baduanjin reduces the risk of falls may be achieved by modulating vestibular nerve pathways. Studies have shown that movement activates areas of the anterior temporal lobe, middle temporal gyrus, superior temporal gyrus, insula, cingulate gyrus, caudate nucleus, and corpus callosum. Exercise training in T2-weighted images modulates functional connectivity in core brain regions of the vestibular neural pathway [18]. In addition, exercise slows neurodegeneration in the cerebellum, activates cortical neural circuits in the cerebellum, and assists the vestibular pathway in the precise localization of movements [19]. Previous studies have suggested that MCI patients have reduced functional connectivity in the right middle temporal gyrus, right parietal limbic angular gyrus, and right anterior cingulate gyrus and gyrus after Baduanjin training. In the brisk walking group, MCI patients had significantly increased functional connectivity in the right inferior parietal limbic angular gyrus and decreased functional connectivity in the right middle temporal gyrus relative to the health education group [20]. Another study showed an increase in gray matter volume in the right hippocampus and an increase in resting-state functional connectivity between the hippocampus and the right angular gyrus in MCI patients after Baduanjin training compared to brisk walking training [21].

Some previous studies have focused on the improvement of cognitive function in elderly people with MCI but not on the brain network mechanisms by which Baduanjin affects falls in elderly people with MCI [20–22]. Moreover, due to the distributed nature of vestibular neural networks, although the existence of pathways had previously been identified in the laboratory, there was no precise definition of their extent and localization, and it was not until 2020 that an accurate imaging map of vestibular neural networks was obtained [23]. Therefore, previous studies only revealed that some brain regions of the vestibular neural network were associated with the cognitive intervention effects of Baduanjin but did not systematically and thoroughly investigate the role of this network as a whole in reducing the risk of falls in senior MCI patients.

We intend to carry out a prospective, randomized controlled trial (RCT) to examine the effects and mechanisms of three months of Baduanjin training on the risk of falls in elderly individuals with MCI. The main goal of this trial is to verify the intervention effect of Baduanjin training on the risk of falls in elderly people with MCI and to explore the mechanism of Baduanjin in reducing the risk of falls in elderly individuals with MCI from the perspective of functional connectivity of the vestibular neural network.

We hypothesize that Baduanjin training could reduce the risk of falls in older adults with MCI compared with brisk walking and no specific exercise intervention. With neuroimaging, we hypothesize that differential functional connection changes will be observed between the Baduanjin training and the brisk walking group and the health education group.

## Methods

### Study design

**Fig. 1:**
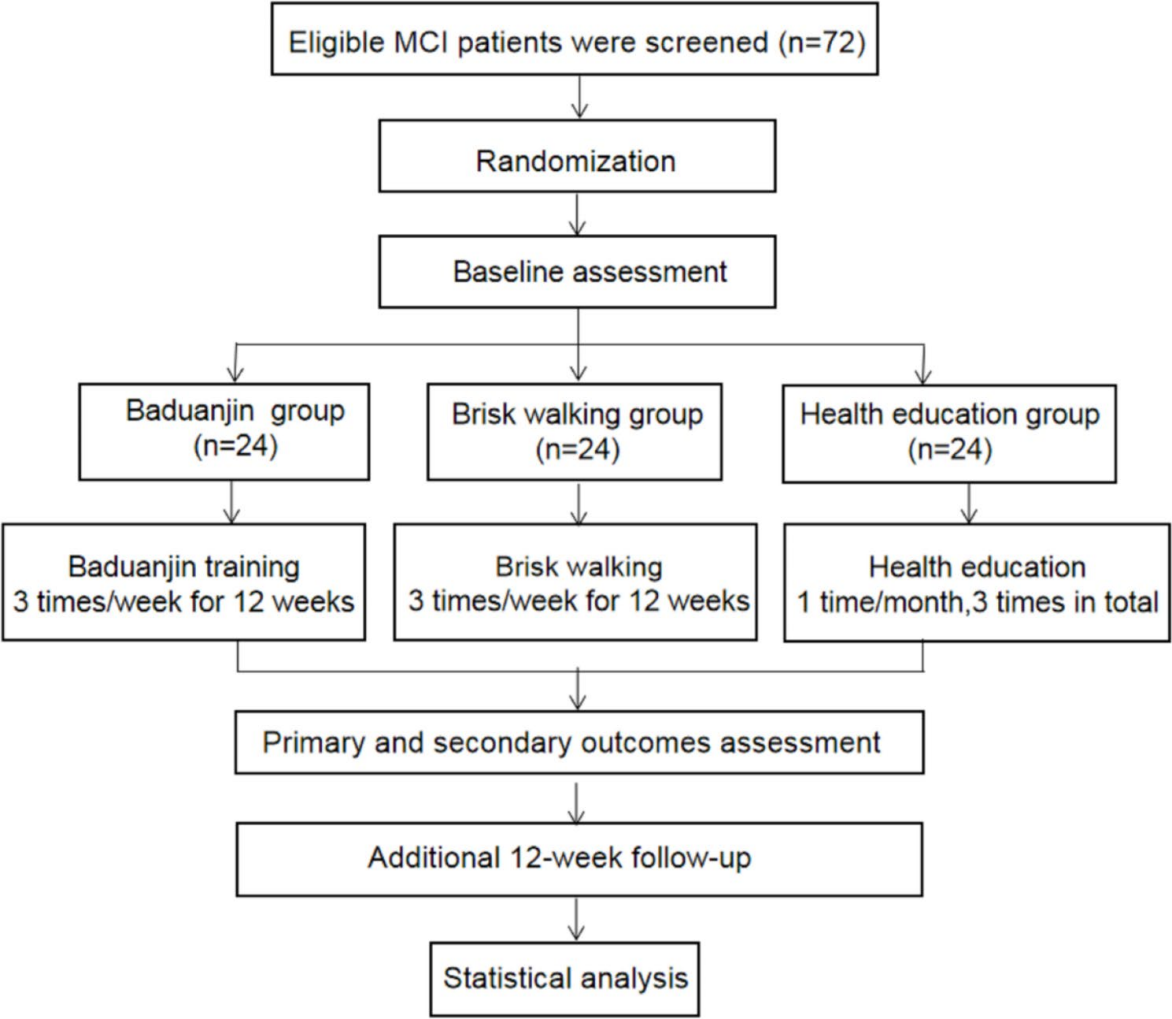
Flow chart of the trial

**Fig. 2:**
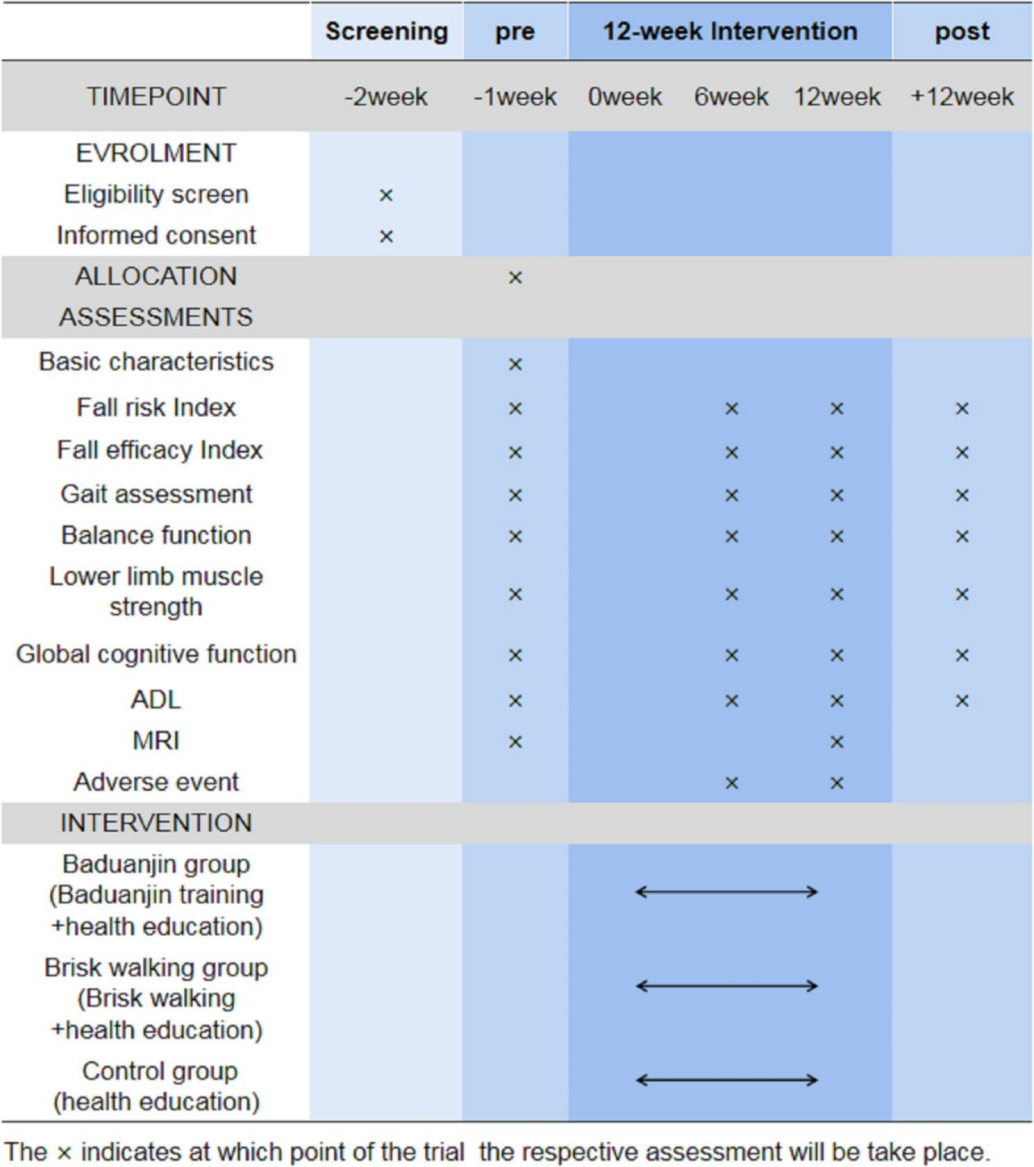
Schedule of enrolment, assessments, and interventions

The design of this study is a three-arm RCT in a 1:1:1 allocation ratio with allocation concealment and assessor blinding. A total of 72 eligible participants will be randomly assigned to the 12-week Baduanjin exercise intervention (60 min per session, three sessions per week), the 12-week brisk walking group(60 min per session, three sessions per week) or the 12-week health education group. In addition to the MRI scans, which will be measured before and after the intervention,other primary and secondary outcomes will be assessed at baseline, 6 weeks, and 12 weeks (at the end of the intervention) and after an additional 12-week follow-up period. The study procedure and outcome assessment schedule for this trial are presented in Figs. 1 and 2.

### Study population

The study population is a community of older adults with MCI. The diagnostic, inclusion, and exclusion criteria for the study sample are as follows.

### Diagnostic criteria

The criteria for MCI are as follows [24]: (1) cognitive impairment confirmed by a patient or insider, or by an experienced clinician; (2) impairment in one or more cognitive domains (memory, language, visuospatial, or executive function); (3) normal activities of daily living; and (4) no dementia.

#### Inclusion criteria

The eligible participants must meet the following criteria for inclusion: (1) diagnosed with MCI; (2) aged 60 years or older; (3)Montreal Cognitive Assessment (MoCA) score of<26points (if the years of education are ≤ 12 years, add 1 point to the score); (4)had no regular physical exercise for at least half a year (regular exercise means exercise with a frequency of at least three times a week and at least 20 min per session);(5)no contraindications to MRI scans; and (6) provided informed consent.

### Exclusion criteria

The exclusion criteria are as follows: (1) patients with hypertension with uncontrolled blood pressure (systolic blood pressure greater than 160 mm Hg or diastolic blood pressure greater than 100 mm Hg); (2) history of alcohol or drug abuse; (3) depression-induced cognitive impairment, Beck Depression Scale > 4 points; (4)cognitive impairment due to other causes such as poisoning or drugs;

(5)history of mental illness (including personality disorder, schizophrenia, etc.), severe aphasia or audio-visual disorders, severe organ failure, history of coronary artery disease, musculoskeletal disorders, or other exercise contraindications; and (6) participation in another clinical study.

### Withdrawal criteria and management

Participants will be allowed or required to withdraw from the study based on the following criteria: (1) major violations of the study protocol; (2) suffering from a serious disease, preventing the continuation of the trial; (3) sufferiing from serious adverse events related to the research during the study period; and (4) voluntary request to be withdrawn from the trial.

#### Recruitment

Participants will be recruited from Dalang Community Center and Xingdong Community Center in Shenzhen city through a combination of online and offline efforts, including internect recruitment, posting posters, sending leaflets, and setting-up onsite recruitment stations. Potentially eligible individuals will first complete a screening by community doctors in the Community Health Service Center to determine their eligibility according to the inclusion and exclusion criteria. The eligible individuals who are interested in participating in the study will have an informed discussion with trained research assistants. The research assistants will obtain written informed consent from individuals willing to participate in the study before starting the baseline assessment [25].

#### Randomization, allocation concealment, and blinding

After the baseline assessment, the eligible participants will be randomly assigned to the Baduanjin group, the brisk walking group, or the health education group. The simple random allocation sequence will be generated using the PLAN procedure of the statistical software SAS V.9.0 and will be managed by an independent research assistant who is not involved in the recruitment, evaluation, and intervention of the participants. The eligible participants will be informed of their allocation result by the independent research assistant via telephone [26]. We cannot blind participants, exercise coaches, or intervention supervisors to the assigned treatment, but outcome evaluators and data statisticians will be blinded to group allocation.

### Intervention

#### Baduanjin group

Based on the health education group, participants in the Baduanjin group will receive 12 weeks of Baduanjin exercise training with a frequency of 3 days a week and 60 min a day [27, 28]. Baduanjin exercise training will be assigned at two community centres (Dalang Community Centre and Xingdong Community Centre in Shenzhen city) with 30–40 individuals per centre. Two professional coaches who have taught Baduanjin for at least 5 years will be hired to guide the training of the trainees. The training scheme of the Baduanjin exercise is planned as previously described in detail and consists of 10 postures [25] (including the preparation and ending postures).

### Brisk walking group

Based on the health education group, participants in the brisk walking group will practise brisk walking under the guidance of the coach before the intervention and will then go home to practise on their own after receiving instruction. The project manager will call weekly before the intervention to urge the participants to train as needed, and the participants will self-report the training time. Subjects in the brisk walking group will engage in 12 weeks of walking training with a frequency of 3 days a week and 60 min a day, consisting of a 15 min warm-up, 40 min of walking training and a 5 min cool-down.The walking training will require subjects to walk 120–140 steps per minute, have a heart rate of 55-75% of their maximum heart rate (206.9 − 0.67*age), meet the requirements of moderate exercise intensity, and feel relaxed or free of dizziness, nausea, and fatigue [20, 29].

### Health education group

The participants in the health education group will not receive any specific exercise training in the research plan, except for fall health education before the intervention, including the factors affecting falls, the hazards of falling, and the commonly used methods of preventing falls. They will be required to maintain their original activity habits. To exclude bias from the excess activity of participants, all participants will be advised not to seek any other regular exercise and will be required to record activity during the trial period according to the previously described method [26].

### Outcome assessment

The variables in this trial will consist of basic information, primary outcomes, and secondary outcomes. The basic characteristics will be measured at baseline (1–2 weeks before randomized allocation). In addition to the MRI scans, which will be measured before and after the intervention,other primary and secondary outcomes will be measured at baseline, in the middle of the intervention period (6 weeks after randomization), at the end of the intervention period (12 weeks after randomization) and after an additional 12-week follow-up period. All primary and secondary outcomes will be assessed by the experienced staff at the People’s Hospital Baoan Shenzhen, who will be blinded to the allocation results of participants. A summary of all planned measurements for this trial is shown in Fig. 2.

### Basic information

Participants’ demographic characteristics (e.g., sex, age, education, marital status, living arrangements, occupation, and socioeconomic status) and history of disease and medication use will be collected by the recruiters using a self-designed questionnaire. The basic MoCA and Beck Depression Inventory will be assessed using the corresponding scales. Baseline measurements will be completed before randomization.

### Primary outcomes

#### Fall risk

The Fall-Risk Self-Assessment Questionnaire(FRQ) is a short, 12-item instrument specifically designed for community-dwelling seniors to assess their own fall risks [30] .The total score can be obtained by summing the total number of points for all “yes” responses (giving 2 points to questions 1 and 5 and 1 point to all other questions). Participants scoring ≥ 4 (maximum score of 14 with a minimum of 0) are considered to be indicated as having a potential fall risk. The scale has been applied to older Chinese adults with good internal consistency (Cronbach’s α = 0.724) [31].

### Secondary outcomes

#### Fall efficacy

The fall efficacy index will be assessed using the Modified Fall Efficacy Scale (MFES), which is a 14-item quantitative analysis of the fear of falling while dressing or engaging in daily activities and a simple analysis of self-presentation efficacy in older adults. The first 9 items of this assessment are indoor activities and the last 5 items are outdoor activities, each with a score from 0 to 10 (0 = no confidence at all; 5 = average confidence; 10 = sufficient confidence), for a total of 11 levels. The cumulative average score for each item is the final score [32].

### Gait assessment

Gait assessment will be assessed using the Tinetti Performance Oriented Mobility Gait Assessment(POMA-G), which includes the following subscales; initiation of gait, step length, step symmetry, step continuity, path, trunk and walking stance.Each subscale is measured as abnormal = 0 or normal = 1; in some cases, adaptive = 1 and normal = 2. The total score was 12, with higher scores indicating better mobility and balance [33].

### Balance function

Balance function will be tested using a static and dynamic balance assessment analyser (model: Bismarck Super Balance), which uses a plantar pressure sensor to accurately map the trajectory of the subject’s plantar weight shift in four positions: bipedal with eyes open, bipedal with eyes closed, unipedal with eyes open, and unipedal with eyes closed. By measuring the total trajectory length (mm), peripheral area (mm^2^), trajectory length per unit area, average centre variation in the X-axis (mm), average centre variation in the Y-axis (mm), and Romberg’s quotient, we analysed the visual adjustment coefficient, balance stability coefficient, and proprioceptive coefficient to assess the degree of visual, vestibular and proprioceptive effects on balance and finally obtained the balance index of the subjects.

The visual adjustment coefficient reflects the role of vision in posture control. The larger the value, the greater the role of vision in posture control. In contrast, in the absence of visual feedback, the balance function of the body will become worse. The balance stability coefficient reflects the stability of the body. The larger the coefficient is, the smaller the degree of shaking and the better the stability of the body.The proprioceptive coefficient reflects the function of proprioception in posture control. The larger the coefficient, the stronger the proprioceptive posture adjustment ability and the easier it is to maintain balance.

#### Lower limb muscle strength

Lower limb muscle strength will be assessed using the 5 Times Sit-to-Stand (5STS) and the Thirty-Second Sit-to-Stand Test to determine the presence or absence of lower extremity muscle weakness. A chair will be adjusted to the height of each person and will be fixed to the wall.After a researcher explained the requirements and precautions to the participant, the participant was asked to sit with his or her feet exactly flat on the ground and his or her upper limbs folded across his or her chest and then to stand up all the way and sit down again without using his or her arms.The participant will be asked to repeat this sit-to-stand action five times as quickly as possible, and the time taken to complete the five repetitions will be recorded.Then, the participant will be asked to repeat as many of the sit-to-stand actions as possible in 30 s, and the maximum number completed will be recorded. The minimum value of three trials of the 5STS and the maximum value of two trials of the 30STS will be considered the participant’s score. Once the participant is unable to perform the motion or asks to suspend the test, the test will be terminated [34, 35].

### Cognitive function

Global cognitive function will be measured using the MoCA(Fuzhou version) scale, which is a cognitive screening instrument created and validated to detect mild cognitive impairment [26]. MoCA is a brief (approximately 10 min) test that evaluates visuospatial/executive functions, naming, verbal memory registration, learning, attention, abstraction, 5 min delayed verbal memory, and orientation with a total score of 0–30 (a higher score equates to a better function).

#### Activities of daily living

Activities of daily living(ADL) will be assessed using the Barthel Index(BI), which is a widely used standard scale for assessing functional disability in ADL.The BI contains 10 projects with a total of 100 points, and the higher the score is, the better the daily life self-care ability.

#### MRI scan

To further explore the potential mechanism of Baduanjin in reducing the risk of falls in patients with MCI, this study will collect participants’ resting-state MRI data before and after the intervention. The MRI scan will be conducted in the key laboratory for magnetic resonance and multimodality imaging of Guangdong province. A Siemens Prisma 3.0 T MRI system and Siemens 64-channel head and neck coil will be used for acquiring fMRI data. The resting fMRI scanning parameters are listed as follows: TR = 2000 ms, TE = 30 ms, flip angle = 90°, slice thickness = 3.6 mm, FOV = 230 mm×230 mm, matrix = 64 × 64, voxel size = 3.6 × 3.6 × 3.6 mm3, number of slices = 37, axial slices = 35, ad phases per location = 240.

#### Safety measurements

Participants will be monitored during the intervention period for the occurrence of adverse events. Any adverse events, including exercise injury and falls, will be detailed and reported by the research assistant to the research group using an adverse event case report form (CRF). The causality concerning the Baduanjin intervention and the severity of adverse events will be evaluated. Serious adverse events will be reported to the ethics committee.

### Sample size

The results of a previous systematic review showed that the standardized mean difference of the fall risk of the elderly with Baduanjin exercise intervention was − 0.59 [-0.83, -0.35] [36], which was used as a reference to calculate the sample size of the effect size of this study. The significance level will be set at α = 0.05, and the power will be 0.80. Using Gpower 3.9 software, we calculated that a sample size of at least 20 patients per group is needed, and considering a 20% drop-out rate, which means 24 patients per group, the total sample size required for this study is 72 cases.

### Statistical analysis

The data of this study will be statistically analysed using SPSS 26.0 software. All statistical tests will be performed using a two-sided test with an α value of 0.05, and we will consider the test results statistically significant when the resulting p value is ≤ 0.05.Count data will be described using frequencies, percentages, or composition ratios. The continuous data will be described by mean ± standard deviation or median. This study will include multiple imputation for missing data and an intention-to-treat analysis. One-way ANOVA will be used for data satisfying a normal distribution, teh rank sum test will be used for those not satisfying a normal distribution, and the chi-square test will be used for count data.

Primary outcomes will be further analysed with the mixed-effect linear model with restricted maximum likelihood to determine the interaction effect between group and time. In this model, the independent variables are group (Baduanjin group, brisk walking group, and health education group), time (different measurement time points), sex, age, education level and other confounding factors, all of which will be analysed as covariates.

Preprocessing of the fMRI data was performed using the Functional Connectivity Toolbox (CONN) pipeline version 21a (www.nitrc.org/projects/conn, accessed on 5 September 2022). The preprocessing included slice-timing correction, segmentation, normalization, smoothing (Gaussian kernel = 8 mm), and bandpass filtering (0.008 to 0.08 Hz) [37]. We will implement a functional connectivity analysis of the corticocortical vestibular network based on the region of interest (ROI). The selection and specific analysis method of the corticocortical vestibular network ROI is similar to that of a previous study which was conducted in 2022 [38].

Adverse effects (AEs) will be analysed using the χ^2^ test or Fisher’s exact test. If formal statistical analyses between groups cannot be performed due to the lack of power, AEs will be tabulated and summarized using descriptive statistics.

## Data collection and management

Data will be collected by the outcome assessors using the CRF. Then, research assistants will transcribe the CRF into an electronic data acquisition system (EDC).

This is a free Research Manager (ResMan, http://www.medresman.org) provided by the China Clinical Trial Registry and meets the available standards for security.

The research data will be stored in the EDC system in a separate password-protected location [25].

### Ethical issues

This study protocol is in accordance with the Helsinki Declaration. Ethics approval was obtained from the Medical Ethics Committee of the People’s Hospital Baoan Shenzhen(Approval no. BYL20210905). All participants will provide voluntary written informed consent after fully discussing potential benefits and risks before participating.

### Dissemination

The study protocol has been registered and can be obtained through the Chinese Registry website (registered on ChiCTR.org with the identifier ChiCTR2200057520). The results of the study will be presented at local, national, or international conferences about cognitive rehabilitation and will be submitted as manuscripts to peer-reviewed journals. The main findings of this study will also be shared with all participants and disseminated to researchers, health service providers, health care professionals, and the public through courses, demonstrations, and the internet regardless of the magnitude or direction of the effects.

## Discussion

Traditional Chinese medicine theory includes the belief that Baduanjin has the effect of “unifying body and mind”. Existing research suggests that Baduanjin exercise can reduce the risk of falls for participants, and at the same time [39]_,_ it has a continuous improvement effect on cognitive function in older adults with MCI [28]. Baduanjin intervention is safe, effective, simple, and easy to implement, and it is suitable for extensive development of fitness in MCI patients. In addition, a few rigorous methods will be used in this trial to control for bias, including randomization, parallel control, and blinding of statisticians and outcome assessors. We will also employ certified physical exercise instructors as Baduanjin exercise coaches. The Baduanjin intervention will include a specific training regimen (60 min per day, 3 days per week for 12 weeks) to clarify its association with fall risk. Therefore, extensive outcome assessment at multiple levels, including through the Fall Risk Self-Assessment Scale, dynamic and static balance assessment, and MRI, will provide evidence that Baduanjin training reduces the risk of falls in MCI patients by modulating the functional connectivity patterns of vestibular neural networks.The findings should help improve balance function and reduce the risk of falls in MCI participants and should be applicable to older adults with MCI.

In conclusion, the effects of a Baduanjin intervention on falls and balance function will be thoroughly evaluated in this study for the first time through a randomized controlled experiment. If the experiment is successfully completed and shows meaningful outcomes, this research will help provide a scientific basis for Baduanjin intervention for falls in elderly individuals with MCI, and also provide fresh proof for the promotion and use of conventional exercise treatment.

### RCT status

The study implementation period starts in March 2022 and ends in March 2024.

### Provenance and peer review

Not commissioned; externally peer reviewed.

## Data Availability

Not applicable. No data have been generated. Raw data sharing will be available after 31 March 2024 by contacting the corresponding author.
